# HB-EGF-induced IL-8 secretion from airway epithelium leads to lung fibroblast proliferation and migration

**DOI:** 10.1186/s12890-021-01726-w

**Published:** 2021-11-06

**Authors:** Yanyu Li, Guomei Su, Yu Zhong, Zhilin Xiong, Tong Huang, Jingyun Quan, Jiewen Huang, Xiaoxia Wen, Chaole Luo, Weilin Zheng, Jinfeng Chen, Junfen Cheng, Weimin Yao, Tianwen Lai

**Affiliations:** 1 Department of Respiratory and Critical Care Medicine, The Affiliated Hospital of Guangdong Medical University, Zhanjiang, 524001 China; 2 Department of Respiratory and Critical Care Medicine, The Second Affiliated Hospital of Guangdong Medical University, Zhanjiang, 524001 China

**Keywords:** HB-EGF, Lung fibrosis, IL-8, Airway epithelium

## Abstract

**Background:**

We have reported that heparin-binding epidermal growth factor (HB-EGF) is increased in patients with chronic obstructive pulmonary disease (COPD) and associated with collagen deposition, but the mechanisms remain unclear. In the present study, we aimed to investigated the inflammatory cytokines secreted by bronchial epithelial cells following exposure to HB-EGF that promoted proliferation and migration of human lung fibroblast.

**Methods:**

HB-EGF–induced inflammatory cytokines were assayed in two airway epithelial cells (primary human bronchial epithelial cells [HBECs] and BEAS-2B cells). Moreover, the culture supernatants derived from HB-EGF-treated HBECs and BEAS-2B cells were added to human primary lung fibroblasts. The effect of culture supernatants on proliferation and migration of fibroblasts was assessed.

**Results:**

IL-8 expression was significantly increased in bronchial epithelial cells treated with HB-EGF, which was at least partially dependent on NF-kB pathways activation. HB-EGF–induced IL-8 was found to further promote lung fibroblasts proliferation and migration, and the effects were attenuated after neutralizing IL-8.

**Conclusions:**

These findings suggest that HB-EGF may be involved in the pathology of airway fibrosis by induction of IL-8 from airway epithelium, subsequently causing lung fibroblasts proliferation and migration. Thus, inhibition of HBEGF and/or IL-8 production could prevent the development of airway fibrosis by modulating fibroblast activation.

**Supplementary Information:**

The online version contains supplementary material available at 10.1186/s12890-021-01726-w.

## Background

The airway epithelium plays an important role in first line of defense mechanisms through physical and chemical barriers. The peribronchiolar collagen and subepithelial fibrosis are major causes for airway remodeling and airflow limitation. Previous studies have shown that aberrant extracellular matrix (ECM) deposition drives airway inflammation, repair, cell migration, and proliferation. ECM is a key structural change in airway wall remodeling [1, 2]. Among lung resident cells, fibroblasts play a critical role in peribronchiolar collagen and subepithelial fibrosis. Fibroblast-mediated airway ECM protein and collagen deposition are important caused of airway limitation [2]. However, the pathological mechanism involved in airway fibrosis is still unclear.

Heparin-binding epidermal growth factor (HB-EGF) is a member of the epidermal growth factor (EGF) superfamily [3]. HB-EGF is a potent chemotactic factor for fibroblasts, epithelial, smooth muscle cells. Wang et al. demonstrated that the expression of HB-EGF is related to the thickening of airway smooth muscle (ASM) and induces the migration and proliferation of ASM in vitro [4]. Hirota et al. indicated that histamine may induce airway remodeling via the epithelial-derived HB-EGF [5]. Allahverdian et al. suggested that IL-13-reparative effect on AEC are mediated via HB-EGF [6]. Our previous study has shown that HB-EGF expression was significantly increased in COPD patients and related to airway collagen deposition [7]. However, the biology of HB-EGF in airway fibrosis remains unclear. Further studies are needed to explore the role of HB-EGF–mediated inflammation and structural changes of remodeling.

In the present study, we assessed the potential role of HB-EGF–mediated IL-8 secretion from two airway epithelial cells (primary human bronchial epithelial cells [HBECs] and BEAS-2B cells) in proliferation and migration of primary normal human lung fibroblasts.

## Materials and methods

### Cell culture

Two nontumorigenic, immortalized airway epithelial cells (primary HBECs and BEAS-2B cells) were used in our study. The normal primary HBECs purchased from Lonza (Walkersville, MD) and BEAS-2B cells were obtained from cell banks of the American Type Culture Collection (ATCC). Both HBECs and BEAS-2B cells were cultured in RPMI 1640 containing 10% fetal bovine serum (FBS) at 37 °C, 95% humidity and 5% CO_2_ atmosphere.

In order to obtain conditioned media (CM), HBECs and BEAS-2B cells and were treated with 100 ng/ml recombinant human HB-EGF protein (rhHB-EGF, PeproTech, USA) for 1 h. In our previous study, we have found that HB-EGF can promote the proliferation and phenotypic transformation of primary lung fibroblasts [7]. In order to remove the influence of rhHB-EGF itself on primary lung fibroblasts, we chose 1 h after HB-EGF intervention to change the cell culture supernatant (medium) and the changed medium was collected after 23 h, namely CM of BEAS-2B cell treated with HB-EGF (HB-EGF–BEAS-2B-CM) and CM of HBECs treated with HB-EGF (HB-EGF–HBE-CM). IL-8 of HB-EGF–BEAS-2B-CM and HB-EGF–HBE-CM was neutralized by anti–IL-8 antibody (Abcam, Cambridge, UK) to deplete IL-8.

Primary normal human lung fibroblasts were isolated from lung tissue obtained from donors undergoing resection for localized lung carcinoma who gave informed consent, as described in our previous report [2]. Exclusion criteria were as follows: previously received radiotherapy or chemotherapy, respiratory infection within 4 weeks, history of other cancer. Specimens were dissected at a distance of ≥ 5 cm away from the tumor (avoiding areas involving tumors). The characteristics of study subjects are shown in Additional file 4: Table S1. Written informed consent was obtained from all participants. Study protocols were approved by the Ethics of Research Committee of the Affiliated Hospital of Guangdong Medical University.

### ELISA assays

The expression of IL-8, IL-1β, and IL-6 were measured using an ELISA kit (Abcam) with detection limits of 1.8 pg/ml, 5.46 pg/ml, and 1.6 pg/ml, respectively. The levels of IL-8, IL-1β, and IL-6 were assayed by treatment of BEAS-2B cells and HBECs with recombinant HB-EGF. IL-8 depletion of cell supernatant was also confirmed by IL-8 ELISA assay. For experiments employing inhibitors, BEAS-2B cells and HBECs pretreated with 5 μM NF-κB inhibitor (Bay 11-7082, Beyotime Biotechnology) for 1 h and then treated with HB-EGF to assess the levels of IL-8.

### Isolation of nuclear and cytoplasmic fractions

Preparation of nuclear and cytoplasmic fractions was performed using nuclear and cytoplasmic protein extraction kit (Beyotime Biotechnology) by following the manufacturer’s instructions. Briefly, after stimulating with HB-EGF, BEAS-2B cells and HBECs were trypsinized and washed with PBS to remove trypsin by centrifugation at 1000 rpm/min for 5 min. Cell pellets were suspended in cytoplasmic extraction reagent A for 15 min and then added cytoplasmic extraction reagent B for 1 min. Pellets would be centrifuged again at 12,000*g* for 5 min, and supernatant (cytoplasmic fraction) was collected. The pellets would be suspended in nuclear extraction reagent for 30 min, centrifuged at 12,000*g* for 10 min, and the supernatant (nuclear fraction) was collected. Measuring the concentration of nuclear protein and plasma protein with Enhanced BCA Protein Assay Kit (Beyotime Biotechnology) and adding with SDS-PAGE Sample Loading Buffer (Beyotime Biotechnology), samples were boiled for 15 min before further Western blot analysis as described above.

### Western blot analysis

Nuclear and cytoplasmic lysates containing equal amounts of protein (25 μg) were equally loaded on 12% SDS–polyacrylamide gel and transferred to PVDF membranes (Millipore) using a Mini-Protean 2 electrophoresis system (Bio-Rad Laboratories). After blocking with 5% skimmed milk in TBS plus 0.1% Tween-20, the membranes were incubated with NF-κB p65 rabbit polyclonal antibody (Proteintech) overnight at 4 °C followed by HRP-conjugated goat anti-rabbit IgG (Trans Gen Biotech). Detection was visualized using an ECL assay kit (Thermo Fisher Scientific, Inc.). Images were subsequently analyzed using Image J software to quantify the protein expression (National Institutes of Health, USA). All blots were repeated in triplicate.

### RT-PCR

Total RNA was isolated with RNAiso Plus (Takara Bio, Inc.) according to the manufacturer’s protocol. ΔCt was used to calculate the differences between the target cycle threshold (Ct) values and the housekeeping gene: ΔCt = [Ct (target)-Ct (reference)]. Sequences of the primers used for amplification are listed in Table 1.

**Table 1:** Primers used for real-time PCR

Genes	Forward	Reverse
*IL-8* (human gene)	ACTGAGAGTGATTGAGAGTGGAC	AACCCTCTGCACCCAGTTTTC
*IL-6* (human gene)	CCTGAACCTTCCAAAGATGGC	TTCACCAGGCAAGTCTCCTCA
*IL-1β* (human gene)	CCAGGGACAGGTATGGAGCA	TTCAACACGCAGGACAGGTACAG
*GAPDH* (human gene)	TGTTGCCATCAATGACCCCTT	CTCCACGACGTACTCAGCG

### Cell proliferation assays

Primary human lung fibroblasts were seeded into 96-well culture plates. After 24 h incubation, fibroblasts were stimulated with control medium, HB-EGF-BEAS-2B-CM, HB-EGF-HBE-CM, HB-EGF-BEAS-2B-CM + IL-8 antibody, HB-EGF-HBE-CM + IL-8 antibody or medium containing rhIL-8 for 24 h or 48 h (2000 or 3000 cell/well, respectively). For inhibitor experiments, the above CM pretreated with Bay 11-7082 (5 μM) for 1 h and then were collected to stimulate human lung fibroblasts for an additional 48 h. Cell proliferation was evaluated with Cell Counting Kit-8 (CCK-8) (Dojindo Molecular Technologies, Inc.) according to manufacturer’s instructions.

### Cell migration assays

Human lung fibroblasts migration was performed using a 24-well migration chamber with an 8 μm pore polycarbonate membrane (Corning Costar). 2 × 10^5^ cells were suspended in 500 μl of serum-free RPMI 1640 and added to the upper chamber. Control medium, HB-EGF-BEAS-2B-CM, HB-EGF-HBE-CM, HB-EGF-BEAS-2B-CM + IL-8 antibody, HB-EGF-HBE-CM + IL-8 antibody (ab) or medium containing rhIL-8 with 10% FBS was placed in the bottom chamber. After migration for 24 h, the cells that pass through the membrane were fixed with absolute methanol and stained with 0.1% crystal violet for 20 min. For inhibitor experiments, the above CM pretreated with Bay 11-7082 (5 μM) for 1 h were placed in the lower chamber, and lung fibroblasts were seeded into the migration assay. A phase-contrast microscope was used at a magnification of × 200 to count the migrated cells in five random fields per chamber. The experiments were repeated in triplicate at least.

### Plasmids

The Dual-Luciferase Reporter Assay System (Beyotime Biotechnology) was used to detected NF-κB reporter activity. The NF-κB luciferase reporter plasmid was purchased from Beyotime Biotechnology (China). The experiment was performed according to manufacturer’s instructions. The results were presented as luciferase activity of the NF-κB luciferase normalized to Renilla luciferase activity.

### Statistical analysis

GraphPad Prism 8.0 (San Diego, USA) was used for data analysis. Data are presented as mean ± SEM of at least three determinations. Significant differences between the means of the two test groups were analyzed by parametric two-tailed Student’s t-test, and one-way ANOVA with post hoc Tukey’s corrections for the comparison of more than two groups. P < 0.05 was considered to indicate a statistically significant difference.

## Results

### HB-EGF induced IL-8 production in HBECs

**Fig. 1:**
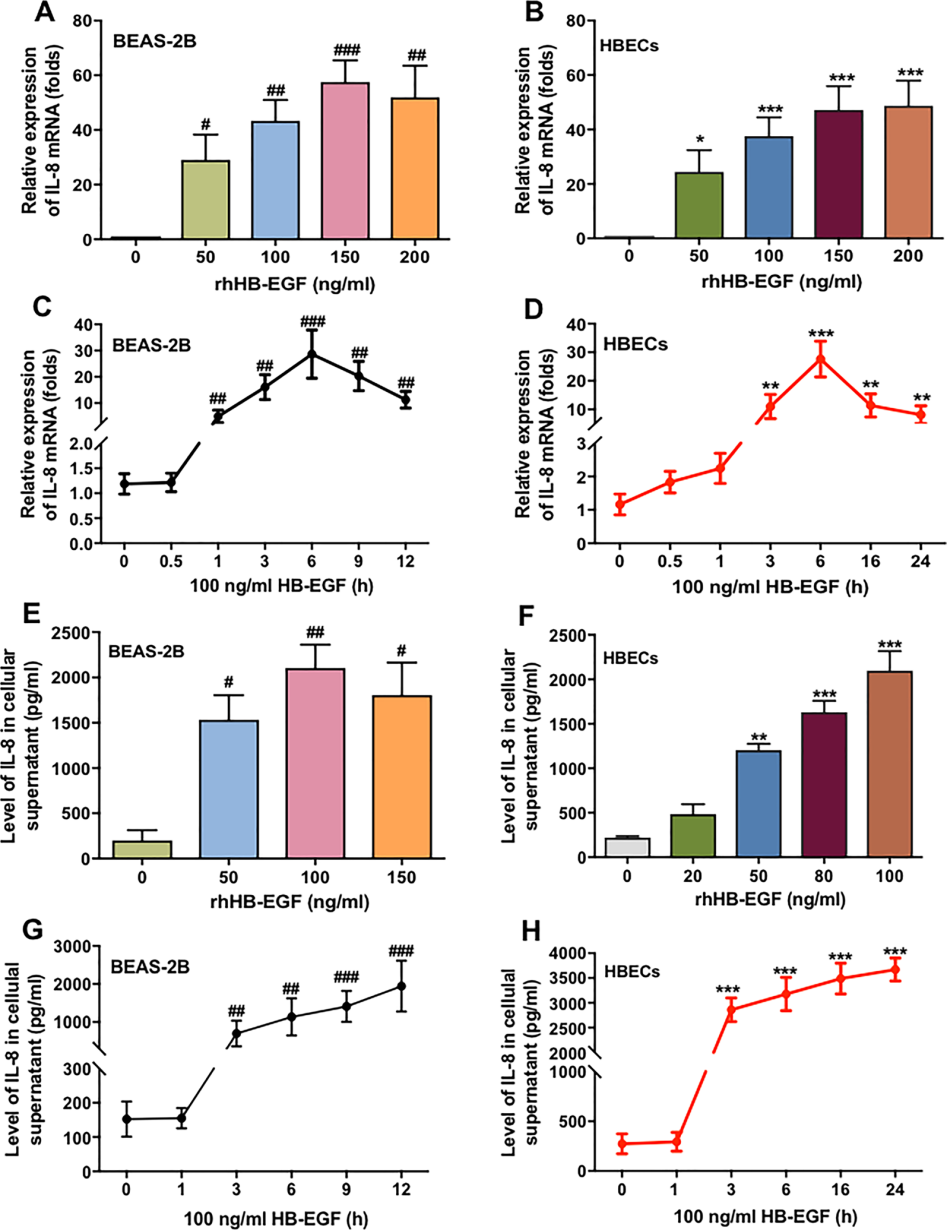
HB-EGF stimulates IL-8 production in BEAS-2B and HBECs. BEAS-2B cells and HBECs were continually stimulated with various concentrations of rhHB-EGF for 6 h (**a** and **b**) or stimulated with 100 ng/ml rhHB-EGF at the indicated time points (**c** and **d**). The cell culture supernatant wasn’t changed and samples was collected at the indicated time points. RT-PCR was conducted to detect the expression of IL-8 mRNA in BEAS-2B and HBE Cs. 6 h was selected based on the IL-8 mRNA expression with a maximum increase at a time point of 6 h (**c** and **d**). ELISA assay was conducted to detect the level of IL-8 secretion in BEAS-2B and HBECs (**e**–**h**). BEAS-2B cells were treated with various concentrations of rhHB-EGF for 6 h (**e**), and HBECs were treated with different concentrations of rhHB-EGF for 24 h (**f**). Both kinds of cells were cultured with 100 ng/ml rhHB-EGF at the indicated time points, with a maximum induction at 12 h in BEAS-2B and 24 h in HBECs (**g** and **h**). Data are from one experiment representative of three independent experiments. Results are expressed as mean ± SEM. ^#^P < 0.05, ^##^P < 0.01, ^###^P < 0.001 versus control of BEAS-2B cells; *P < 0.05, **P < 0.01, ***P < 0.001 versus control of HBECs

The increased inflammatory response in epithelium plays a vital role in the development of chronic inflammatory airway disease. Thus, we first investigated whether HB-EGF induced inflammatory mediators expression in two airway epithelial cells (HBECs and BEAS-2B cells). As shown in Fig. 1a–d, we found that HB-EGF significantly increased the expression of IL-8 mRNA in both BEAS-2B and HBECs in a concentration- and time-dependent manner. HB-EGF also could induce the other inflammatory factors in airway epithelial cells, such as IL-6 and IL-1β (Additional file 1: Fig. S1). However, the increase of other inflammatory mediators were much lower than that of IL-8. As shown in Fig. 1e–h, elevated IL-8 expression in BEAS-2B and HBECs was further confirmed by ELISA. HB-EGF stimulated the release of IL-8 also in a concentration- and time-dependent model (Fig. 1g, h).

### NF-κB pathway was involved in HB-EGF-enhanced IL-8 production in BEAS-2B and HBECs

**Fig. 2:**
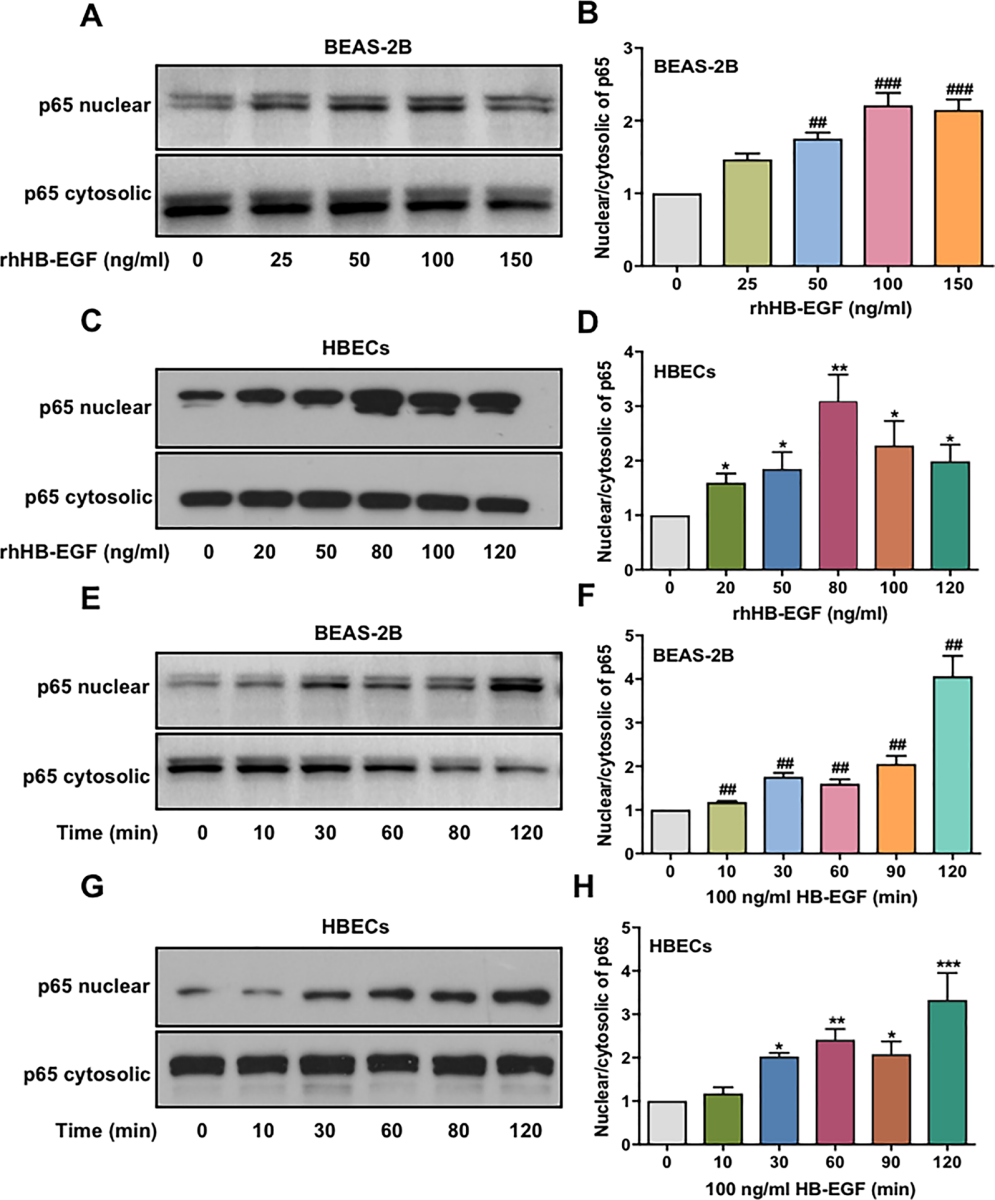
HB-EGF induces activation of the NF-κB pathway in airway epithelial cells. Western blotting was conducted to detect the level of NF-κB p65 (nucleus and cytosol) in BEAS-2B and HBECs. BEAS-2B cells (**a**) and HBECs (**c**) were continually treated with various concentrations of rhHB-EGF for 1 h. The cell culture supernatant wasn’t changed and protein was extracted at 1 h. Further, we used 100 ng/ml rhHB-EGF to stimulate BEAS-2B cells (**e**) and HBECs (**g**) at the indicated time points. The cell culture supernatant wasn’t changed and protein was extracted at the indicated time points. NF-κB p65 (nucleus and cytosol) expression was analyzed using Image J to quantify the protein level. Moreover, the level of nuclear/cytosolic p65 was evaluated NF-κB p65 activation (**b**, **d**, **f**, and **h**). Data are from one experiment representative of three independent experiments. Results are expressed as mean ± SEM. ^##^P < 0.01, ^###^P < 0.001 versus control of BEAS-2B cells; *P < 0.05, **P < 0.01, ***P < 0.001 versus control of HBECs

The NF-κB pathway is well known to play an important role in secretion of inflammatory cytokines such as IL-8 [8]. To assess whether the NF-κB pathway is involved in HB-EGF-induced IL-8 production, the effect of HB-EGF on activation of p65 in BEAS-2B and HBECs was evaluated by Western blotting. As shown in Fig. 2a–d, NF-κB p65 translocate to the nucleus in a dose-dependent manner when HB-EGF stimulates BEAS-2B and HBECs. In addition, we cultured BEAS-2B and HBECs with 100 ng/ml HB-EGF in different time points and observed NF-κB p65 activation in a time-dependent model (Fig. 2e–h).

**Fig. 3:**
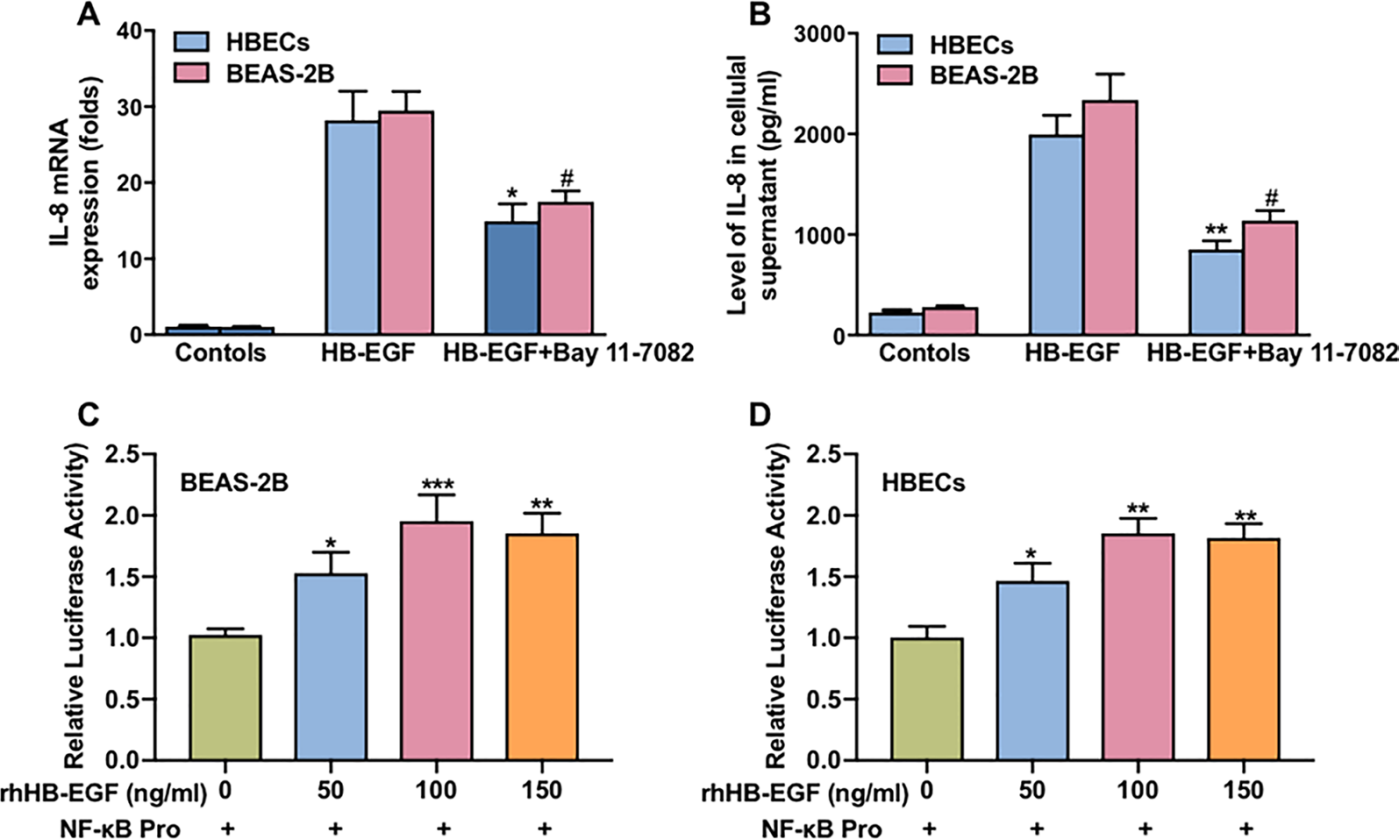
Regulation of IL-8 production by HB-EGF via NF-κB–mediated signaling pathway. The effect of NF-κB inhibitor on HB-EGF–mediated IL-8 mRNA expression (**a**) and IL-8 release (**b**) from BEAS-2B and HBECs. BEAS-2B and HBECs were transfected with the NF-κB-reporter plasmid and then treated with various concentrations of rhHB-EGF for 6 h. Luciferase activity was then measured (**c**, **d**). The cell culture supernatant wasn’t changed and samples was collected at 6 h. Data are from one experiment representative of three independent experiments. Results are expressed as mean ± SEM. ^#^P < 0.05 versus control of BEAS-2B cells; *P < 0.05, **P < 0.01, ***P < 0.001 versus control of HBECs

To further reveal the role of NF-κB in HB-EGF-enhanced IL-8 production in airway epithelium cells, NF-κB inhibitor (Bay 11-7082) was performed in the experiments. The data showed that treatment of HBECs and BEAS-2B cells with Bay 11-7082, before HB-EGF (100 ng/ml) stimulation significantly reduced the IL-8 mRNA expression and release (Fig. 3a, b). Previous study has shown that the IL-8 gene contains NF-κB binding sites in its proximal region [9]. To elucidate mechanisms of NF-κB in HB-EGF-enhanced IL-8 production in HBECs and BEAS-2B cells, HB-EGF–mediated IL-8 transcription was further performed. Luciferase reporter showed that the luciferase activity of NF-κB was significantly increase in HBECs and BEAS-2B cells exposed to HB-EGF and the peak luciferase activity of NF-κB at a concentration of 100 ng/ml for 6 h (Fig. 3c, d).

These results suggested that HB-EGF–induced translocation of NF-κB to the nucleus, where NF-κB binds to specific promoter sites of IL-8 and activates IL-8 gene transcription.

### The CM of HB-EGF–treated airway epithelium cells promoted proliferation and migration of human lung fibroblasts

**Fig. 4:**
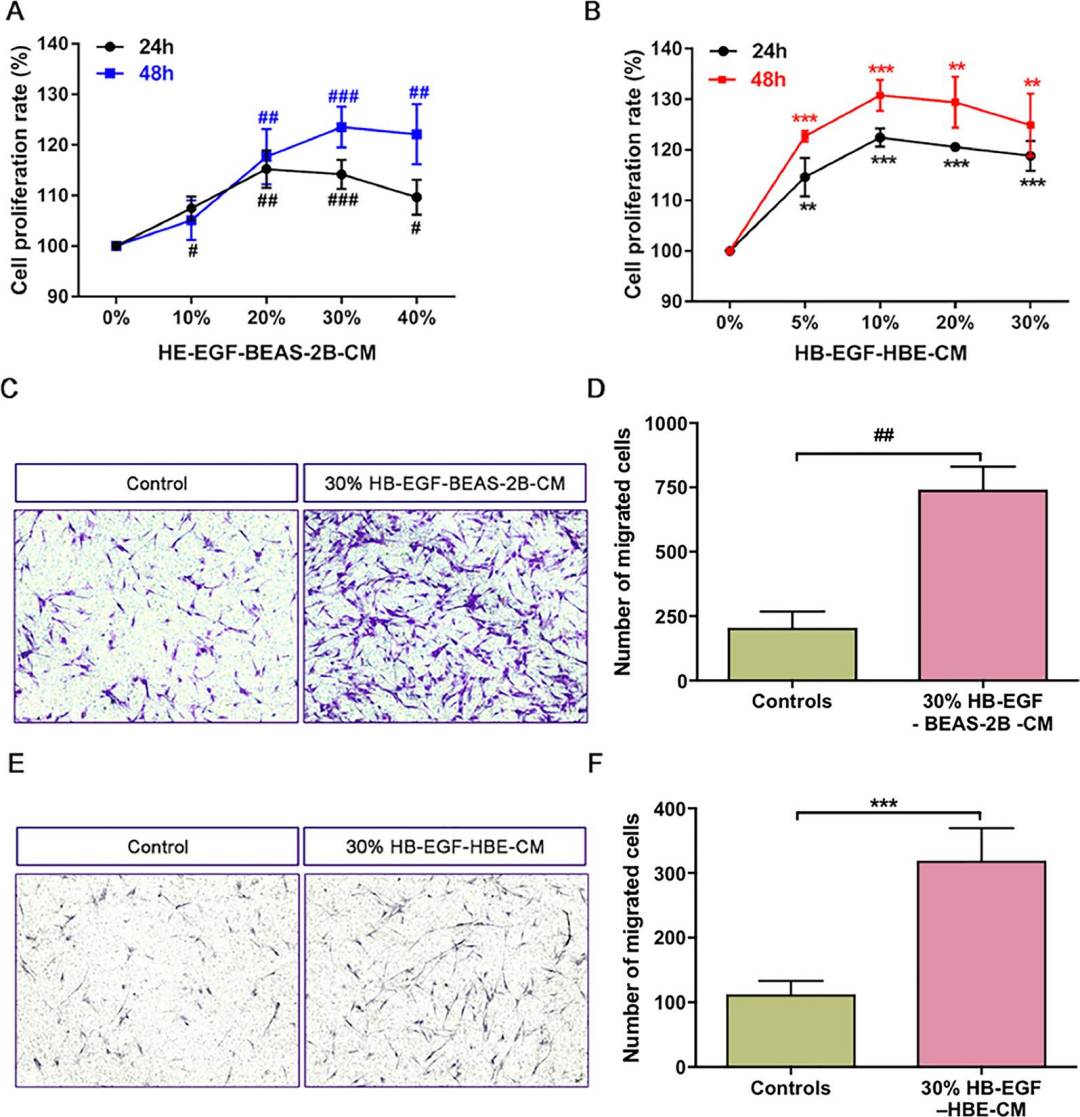
Effect of both HB-EGF-BEAS-2B-CM and HB-EGF-HBE-CM on proliferation and migration of human lung fibroblasts. CCK-8 assay was measured fibroblasts proliferation. Fibroblasts were cultured with various concentrations of HB-EGF-BEAS-2B (**a**) and HB-EGF-HBE-CM (**b**) for 24 h and 48 h. Besides, we used the transwell migration assay to assess the migration capability of fibroblasts. Lung fibroblasts were stimulated with 30% CM of BEAS-2B cells (**c**, **d**) and HBECs (**e**, **f**) for 24 h. Data are from one experiment representative of three independent experiments. Results are expressed as mean ± SEM. ^#^P < 0.05, ^##^P < 0.01, ^###^P < 0.001 versus control medium of BEAS-2B cells; **P < 0.01, ***P < 0.001 versus control medium of HBECs

Active fibroblasts mediated airway fibrosis has been shown to contribute to airway remodeling [1, 2]. We harvested the conditioned media (CM) of BEAS-2B cells and HBECs. Then we cultured human primary lung fibroblasts with CM to detect the effects of CM on the proliferation and migration of fibroblasts. As shown in Fig. 4a, b, treatment with HB-EGF-BEAS-2B-CM and HB-EGF-HBE-CM in 24 h and 48 h cloud significantly promote fibroblasts proliferation in a concentration-dependent manner. Moreover, we found that both 30% CM of BEAS-2B and HBECs could increase the migration capacity of fibroblasts after stimulating for 24 h (Figs. 3f, 4c).

### IL-8 is a potential factor of HB-EGF–treated airway epithelium cells in promoting proliferation and migration of lung fibroblasts

**Fig. 5:**
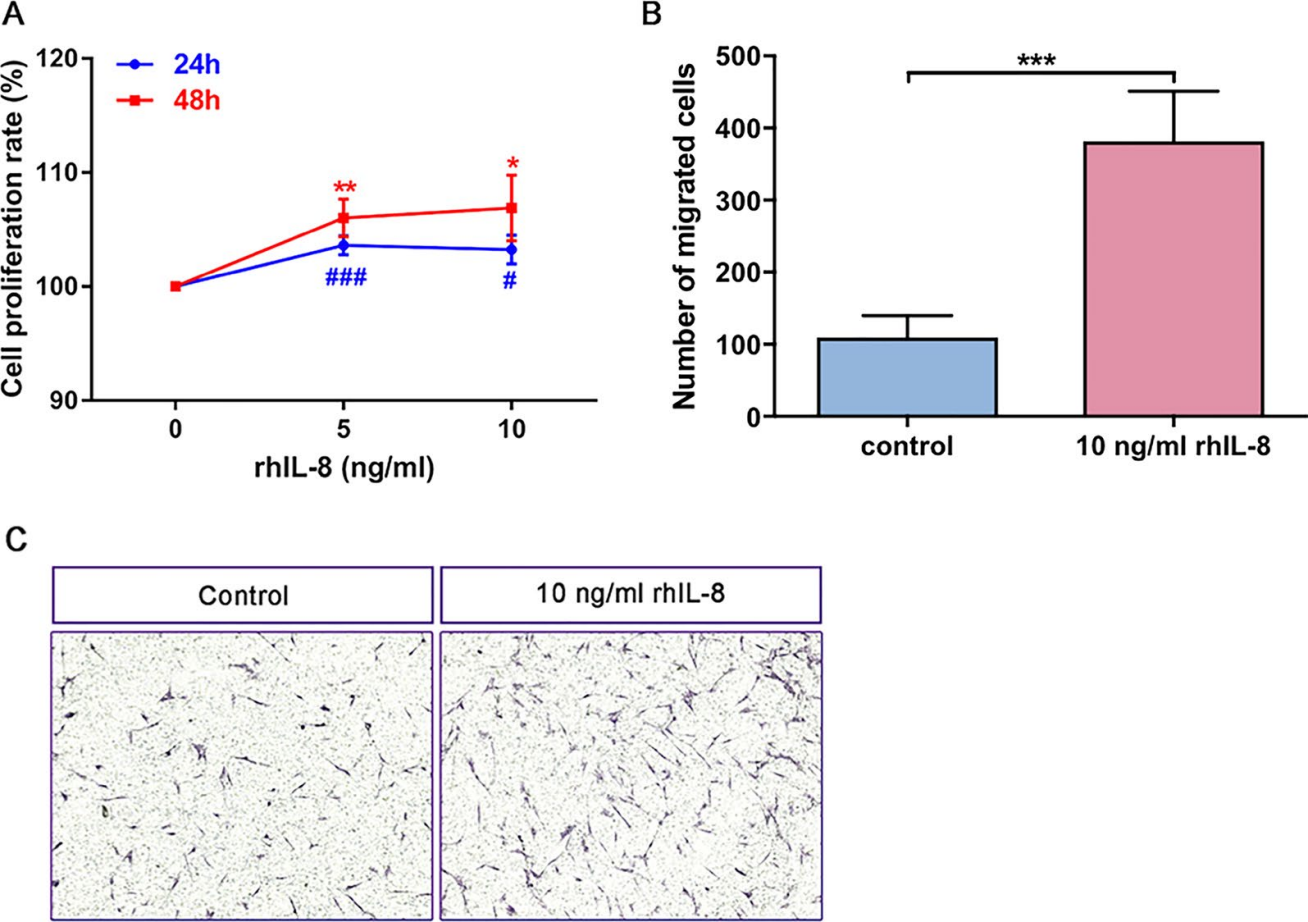
IL-8 promoted proliferation and migration of human lung fibroblasts. CCK-8 assay was used to evaluate fibroblasts proliferation. Lung fibroblasts were treated with different concentrations of combination IL-8 (5 ng/ml and 10 ng/ml) for 24 h and 48 h (**a**). Transwell migration assay was measured the migration capacity of fibroblasts. 10 ng/ml rhIL-8 was used to induce fibroblasts for 24 h (**b**, **c**). Data are from one experiment representative of three independent experiments. Results are expressed as mean ± SEM. ^#^P < 0.05, ^###^P < 0.001 versus control; *P < 0.05, **P < 0.01, ***P < 0.001 versus control

We have shown that HB-EGF significantly increased the IL-8 production in BEAS-2B and HBECs in Fig. 1. Moreover, both CM of BEAS-2B cells and HBECs could promote lung fibroblasts activation. Thus we predicted that IL-8 would be a potential mediator from CM to mediate proliferation and migration of lung fibroblasts. As shown in Fig. 5a, IL-8 promoted proliferation when we treated human lung fibroblasts with various concentrations of rhIL-8 for 24 h and 48 h. Moreover, the migration capability of fibroblasts got heightened as we cultured with 10 ng/ml IL-8 (Fig. 5b, c).

**Fig. 6:**
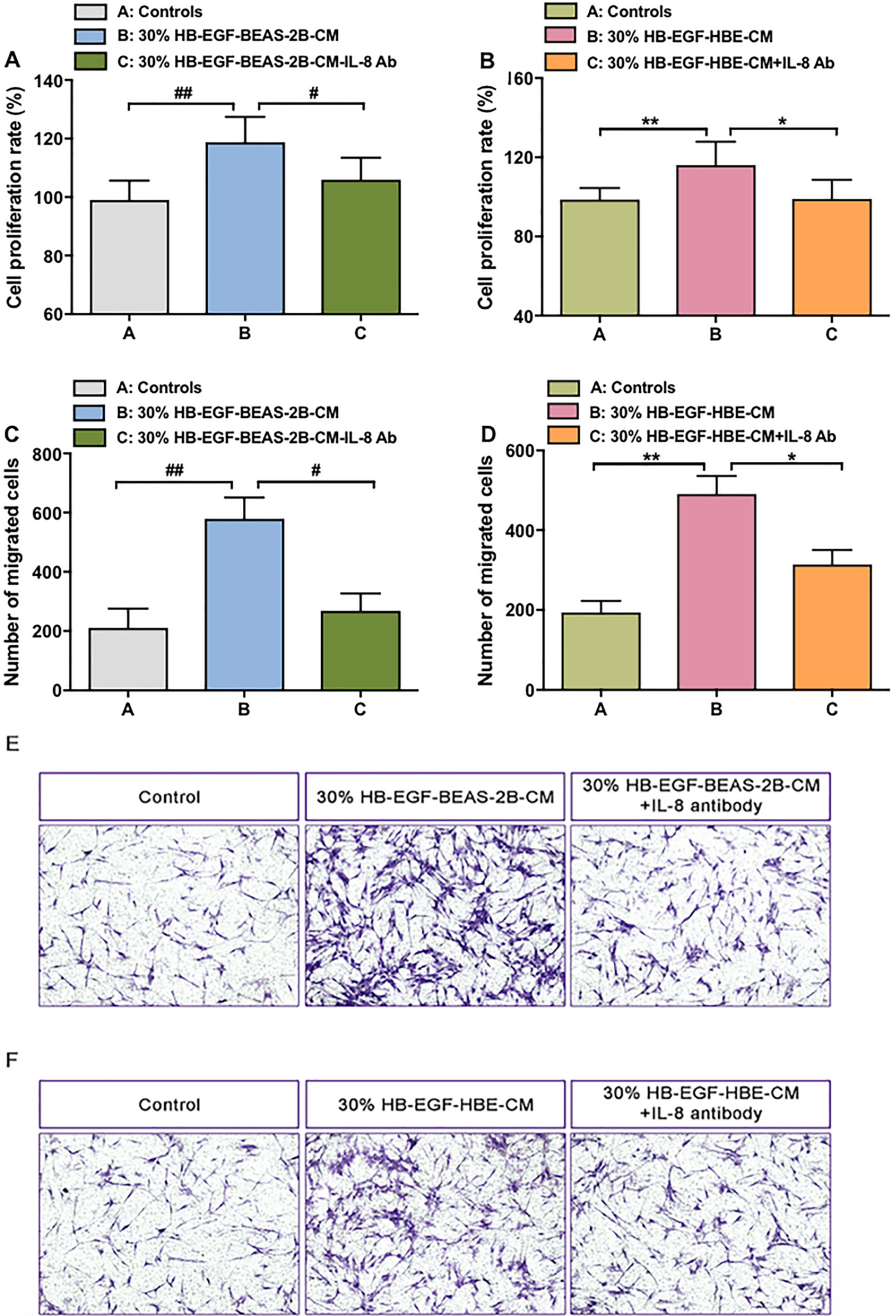
IL-8 is a potential mediator of HB-EGF-BEAS-2B-CM and HB-EGF-HBE-CM in fibroblasts activation. The CCK-8 assay assessed the proliferation of fibroblasts. IL-8 of CM was depleted by antibody (ab). Lung fibroblasts were stimulated with depletion IL-8 CM of BEAS-2B (**a**) and HBECs (**b**) for 24 h. Lung fibroblast migration was measured by transwell migration assay. HB-EGF-BEAS-2B-CM + IL-8 antibody (**c**, **e**) and HB-EGF-HBE-CM + IL-8 antibody (**d**, **f**) was used to culture fibroblasts for 24 h. Data are from one experiment representative of three independent experiments. Results are expressed as mean ± SEM. ^#^P < 0.05, ^##^P < 0.01, ^###^P < 0.001, ^###^P < 0.0001 versus control medium of BEAS-2B cells; *P < 0.05, **P < 0.01, ***P < 0.001 versus control medium of HBECs

To further address the effects of IL-8 on lung fibroblasts activation, we depleted IL-8 from CM of both BEAS-2B and HBECs. The depletion rate of IL-8 from both CM was over 90% (Additional file 2: Fig. S2). HB-EGF-BEAS-CM and HB-EGF-HBE-CM would reduce sharply to stimulate proliferation of lung fibroblasts when they were neutralized by the specific antibody of IL-8 (Fig. 6a, b). Similarly, increased migration capability of fibroblasts by CM of BEAS-2B and HBECs was eliminated to a large extent once IL-8 was depleted, as shown in Fig. 6c–f.

## Discussion

**Fig. 7:**
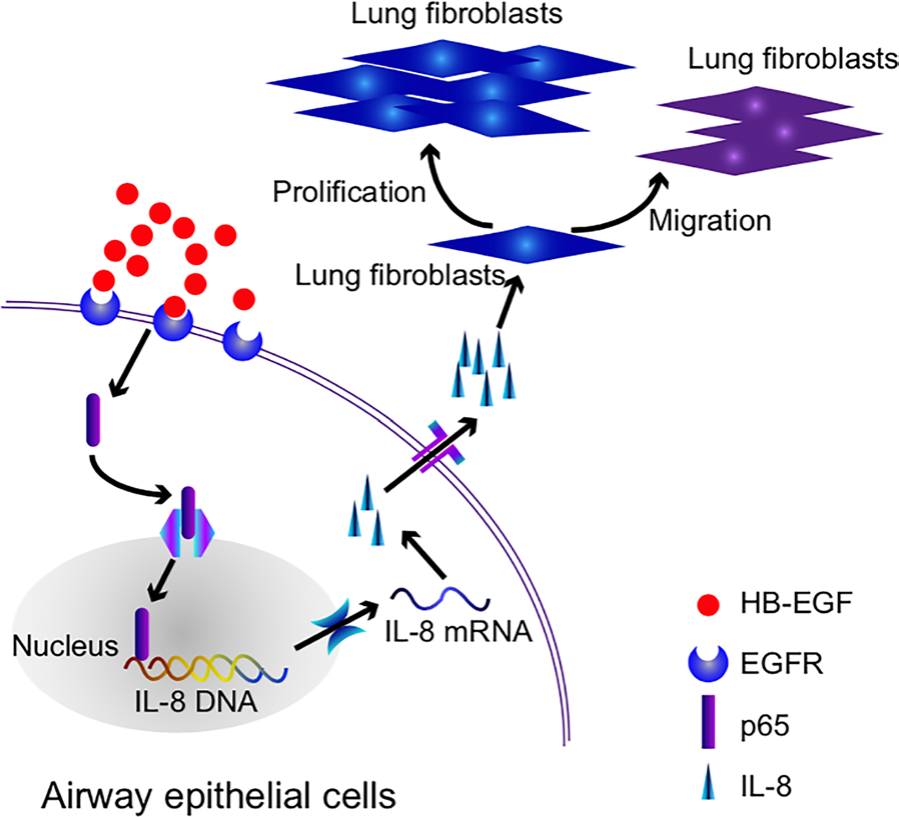
Schematic diagram. Exogenous HB-EGF actives NF-κB pathway by transferring p65 to the nucleus in airway epithelial cells. The activation of NF-κB signaling increases IL-8 production. And then, a large amount of IL-8 is secreted to the extracellular matrix. Under the excessive stimulation of IL-8, the proliferation and migration of lung fibroblasts are activated

To the best of our knowledge, this is the first study to assess the interaction of airway epithelium and lung fibroblasts exposed to HB-EGF. We found that stimulation of airway epithelial cells with HB-EGF leaded to a significant increase of IL-8 release in a time- and concentration-dependent manner. Moreover, NF-κB p65 translocate to the nucleus in a dose-dependent manner when HB-EGF stimulates BEAS-2B and HBECs. Furthermore, BEAS-2B and HBECs pretreated with inhibitors of NF-kB showed a significant decrease in IL-8 secretion exposed to HB-EGF. In addition, we added the conditioned culture media (HB-EGF–HBECs-CM and BEAS-2B-CM) to human primary lung fibroblasts, which resulted in lung fibroblasts proliferation and migration. However, IL-8–depleted HB-EGF–HBECs-CM and BEAS-2B-CM failed to induce the lung fibroblasts proliferation and migration. Taken together, these data provide evidence of HB-EGF–induced IL-8 production in airway epithelial cells via the NF-kB pathway, and IL-8 released from HBECs or BEAS-2B cells following HB-EGF exposure was observed to further promote the lung fibroblast proliferation and migration. Our current study suggested that HB-EGF may involve in airway fibrosis and remodeling by inducing IL-8 secretion (Fig. 7).

HB-EGF, a member of the EGF superfamily, is a potent chemotactic factor for many cell types such as fibroblasts, epithelial and smooth muscle cells [3, 4]. Previous studies have shown that HB-EGF plays a vital role in the physiological functions and pathological processes such as tissue development, pulmonary hypertension, cardiac hypertrophy, and skin wound healing [10]. Our previous study showed that sputum and lung tissue HB-EGF expression was significantly increased in COPD patients and related to disease severity. Moreover, HB-EGF promoted the EMT process in HBECs and collagen deposition in lung fibroblasts, but the mechanisms remain unclear [2]. In the current study, we further study the effect of cytokines and chemokines secreted from HBECs (HBECs and BEAS-2B cells) exposed to HB-EGF on human fibroblasts. Our preliminary data indicated that HB-EGF may be participant in airway fibrosis and remodeling by induction of IL-8 from airway epithelium, subsequently causing proliferation and migration of lung fibroblast.

Previous studies have demonstrated that significantly increased expressions of IL-1β, IL-8 and IL-6 in bronchoalveolar lavage fluid from patients with pulmonary fibrosis in comparison with controls [11]. The cell count and cytokines (IL-1β, IL-6, and IL-8) of bronchoalveolar lavage (BAL) fluid were also increased in bleomycin-induced lung fibrosis in rat [12]. These data suggested that IL-8, IL-1β, and IL-6 were involved in lung fibrosis. In the present study, we found HB-EGF could induce the other inflammatory factors in airway epithelial cells, such as IL-8, IL-6 and IL-1β. However, the increase of IL-6 and IL-1β were much lower than that of IL-8. The primary function of IL-8 is a chemotactic factor that attracts and activates neutrophils in the site of inflammation [13]. Previous studies have shown that IL-8 expression was increased and enhanced neutrophil infiltration in airways of patients with COPD and asthma [14, 15]. We showed that HB-EGF induced IL-8 production in HBECs, which revealing new signaling pathway for induction of IL-8 in HBECs. Moreover, the induced IL-8 expression not only involves in the neutrophils recruitment in the airway inflammation of lung diseases, but also play an important role in airway fibrosis and remodeling via promoting lung fibroblasts proliferation and migration. Activation of NF-κB is the most crucial step for IL-8 gene transcription, as the promoter of the IL-8 gene contains a potential binding site for NF-κB [16]. We also found that HB-EGF promoted IL-8 production via activation of NF-κB. However, the role of HB-EGF on cytokine/chemokine release may be epithelial cell specific due to we only used the bronchial epithelial cell line in this study. Thus, further studies are required to determine whether the role of HB-EGF on IL-8 production is special to the lung in vivo.

Fibroblast-mediated airway fibrosis play a critical role in airway remodeling in COPD and asthma [17, 18]. Increased collagen deposition promotes cellular processes of migration and proliferation and is the key structural change of airway remodeling. The extent of airway remodeling is related to disease progression and airway remodeling is the most important cause for acceleration of lung function decline [19, 20]. Previous studies have shown that IL-8 is a proinflammatory cytokine for fibroblasts, promotes fibroblasts migration and collagen deposition during wound healing in vivo [20, 21]. However, the effect of IL-8 on HB-EGF–related airway remodeling remains unclear. In this study, we demonstrated that HB-EGF promoted IL-8 production from HBECs and BEAS-2B cells, which in turn enhanced the lung fibroblasts proliferation and migration. These effects were confirmed by direct activation of lung fibroblasts with rhIL-8. Moreover, IL-8–depleted or NF-kB inhibitor-pretreated the conditioned culture media (HB-EGF–HBECs-CM and HB-EGF–BEAS-2B-CM) failed to induce the lung fibroblasts proliferation and migration. Although it seems that the NF-kB inhibitor attenuated the lung fibroblasts proliferation and migration caused by HB-EGF–treated CM in HBECs (HBECs and BEAS-2B cells) via inhibition of IL-8 production, other signaling pathways-related direct effects on activation of lung fibroblasts need to be further explored. Our data may be provide an indirect effect of HB-EGF on lung fibroblasts, which is related to IL-8 secretion from HBECs.

### Limitation

The present study does have some limitations. First, clinical specimens are relatively difficult to obtain and primary lung fibroblasts were only passaged to 3–6 generations for further experiments. Therefore, it is difficult to conduct each experiment on the different donors. It is a very interesting to observe whether there are differences between patients in different states. Future studies should be performed to clarify this issue as it may suggest a target for therapeutic intervention. Second, we have focused on the role of HB-EGF on airway epithelial cells and lung fibroblasts in vitro, the current results warrant further investigation in vivo, especially using HB-EGF knockout (KO) mice.

## Conclusions

We demonstrated that HB-EGF promoted IL-8 production in HBECs through NF-kB pathway, and increased IL-8 resulted in enhanced lung fibroblasts proliferation and migration. These results suggest that HB-EGF may be participate in airway inflammation by induction of IL-8 from airway epithelium, subsequently causing airway remodeling. Thus, inhibition of HBEGF and/or IL-8 production could prevent the development of airway fibrosis by modulating fibroblast activation.

## Supplementary Information

**Additional file 1. Fig. S1**: HB-EGF increases IL-1β and IL-6 expression in BEAS-2B and HBECs. BEAS-2B cells were treated with various concentrations of rhHB-EGF for 6 h, and HBECs were stimulated with different concentrations of rhHB-EGF for 24 h. RT-PCR (A, B) and ELISA (C, D) were conducted to detect the expression of IL-1β and IL-6 mRNA in BEAS-2B and HBECs. Data are from one experiment representative of three independent experiments. Results are expressed as mean ± SEM. ^#^P < 0.05, ^##^P < 0.01 versus control of BEAS-2B cells; *P < 0.05, **P < 0.01, ***P < 0.001, ***P < 0.0001 versus control of HBECs.

**Additional file 2. Fig. S2**: Successful depletion of IL-8 in HB-EGF-BEAS-2B-CM and HB-EGF-HBE-CM. ELISA assay was used to assess the neutralization efficiency of IL-8 specific antibody. Data are from one experiment representative of three independent experiments. Results are expressed as mean ± SEM. ***P < 0.001 versus control.

**Additional file 3.** Uncropped scans of Western Blots.

**Additional file 4. Table S1**: Characteristics of donors for primary fibroblasts isolation.

## Data Availability

The datasets used and/or analysed during the current study are available from the corresponding author on reasonable request.

## Abbreviations

HBECs: Human bronchial epithelial cells
HB-EGF: Heparin-binding epidermal growth factor
COPD: Chronic obstructive pulmonary disease
ASM: Airway smooth muscle
