# Supplementary figures and images for: HB-EGF-induced IL-8 secretion from airway epithelium leads to lung fibroblast proliferation and migration
Source: BMC Pulm Med. 2021 Nov 6;21:347. doi: 10.1186/s12890-021-01726-w (PMC8572483; doi:10.1186/s12890-021-01726-w)

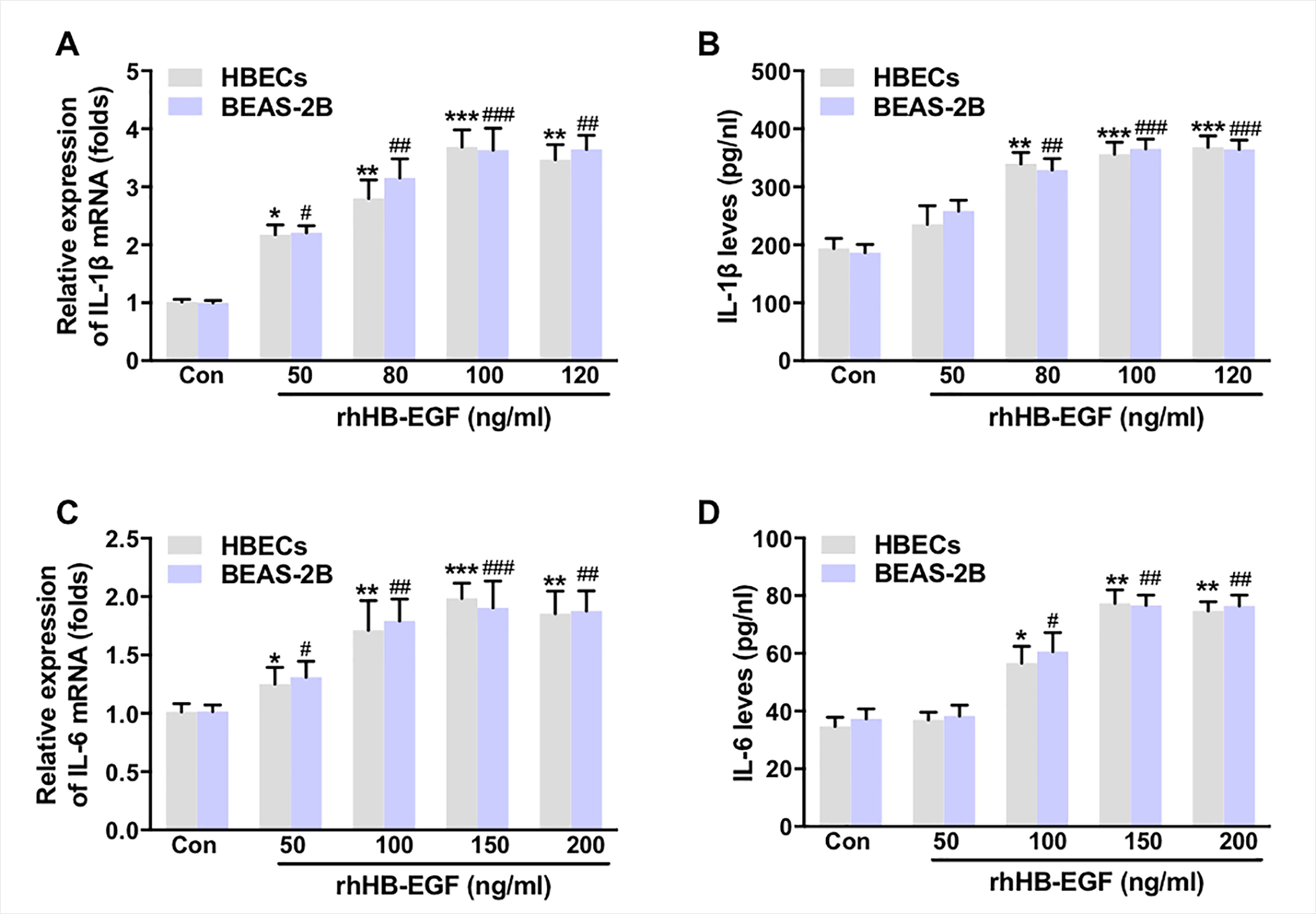

Supplement: Supplementary file 1 — Additional file 1. Fig. S1: HB-EGF increases IL-1β and IL-6 expression in BEAS-2B and HBECs. BEAS-2B cells were treated with various concentrations of rhHB-EGF for 6 h, and HBECs were stimulated with different concentrations of rhHB-EGF for 24 h. RT-PCR (A, B) and ELISA (C, D) were conducted to detect the expression of IL-1β and IL-6 mRNA in BEAS-2B and HBECs. Data are from one experiment representative of three independent experiments. Results are expressed as mean ± SEM. #P < 0.05, ##P < 0.01 versus control of BEAS-2B cells; *P < 0.05, **P < 0.01, ***P < 0.001, ***P < 0.0001 versus control of HBECs. [file 12890_2021_1726_MOESM1_ESM.tiff]

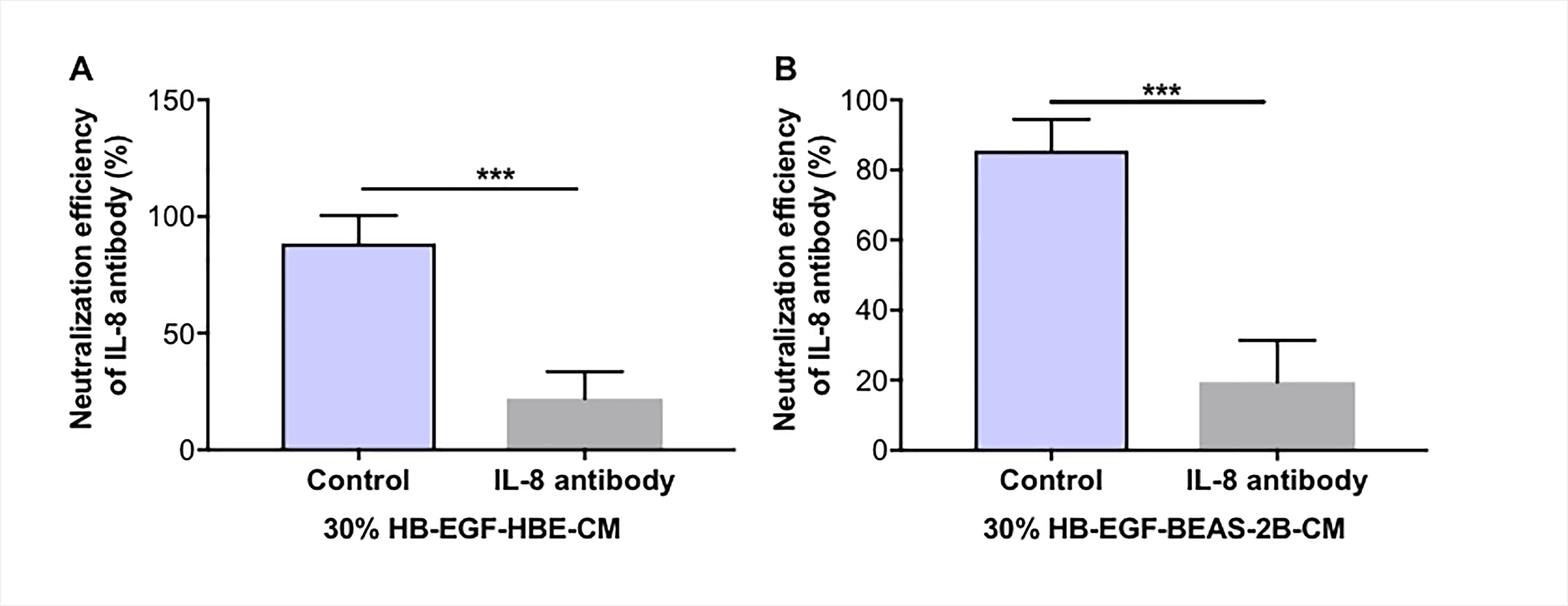

Supplement: Supplementary file 2 — Additional file 2. Fig. S2: Successful depletion of IL-8 in HB-EGF-BEAS-2B-CM and HB-EGF-HBE-CM. ELISA assay was used to assess the neutralization efficiency of IL-8 specific antibody. Data are from one experiment representative of three independent experiments. Results are expressed as mean ± SEM. ***P < 0.001 versus control. [file 12890_2021_1726_MOESM2_ESM.tiff]

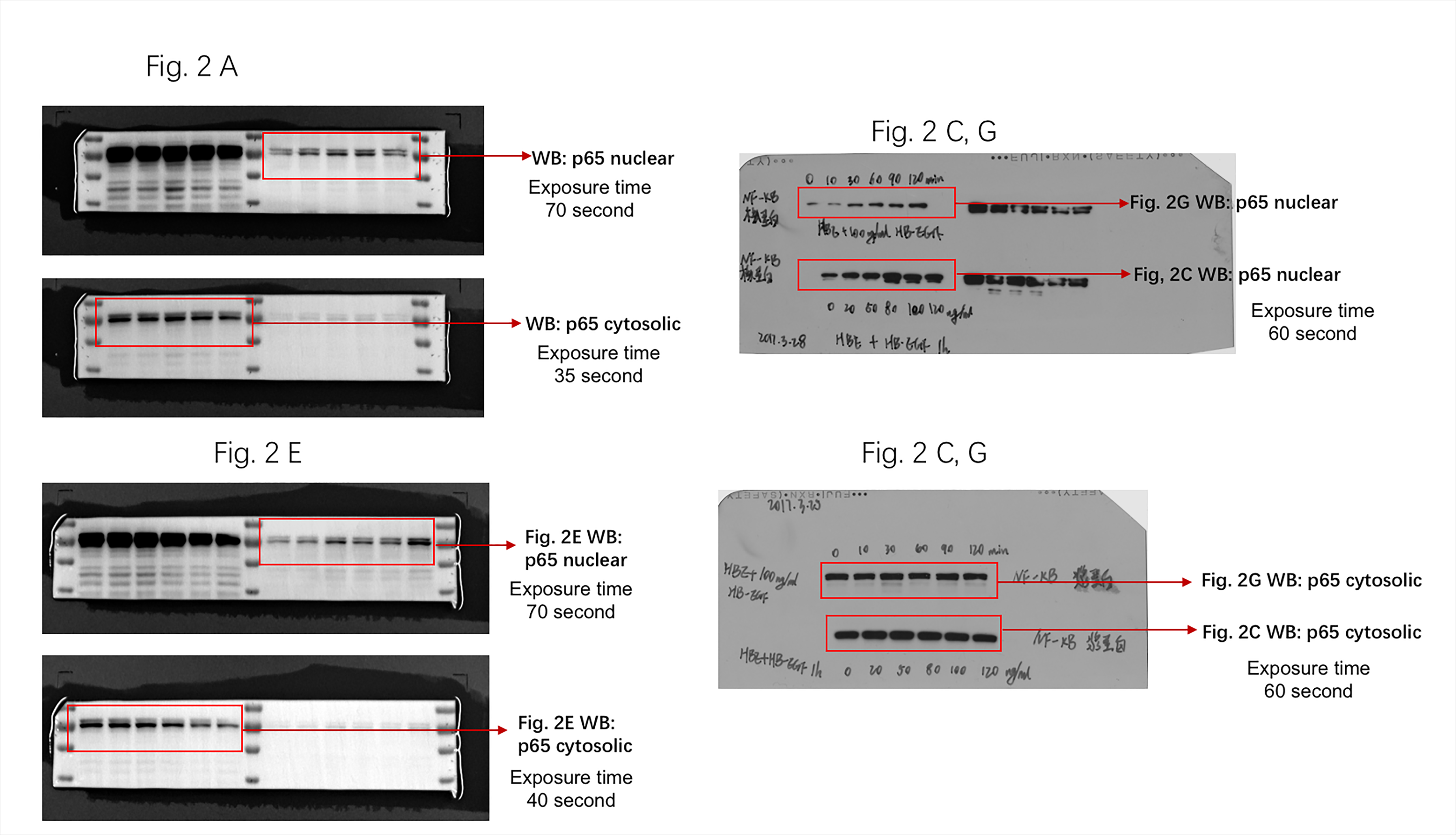

Supplement: Supplementary file 3 — Additional file 3. Uncropped scans of Western Blots. [file 12890_2021_1726_MOESM3_ESM.tiff]
