# Supplementary material for: HB-EGF-induced IL-8 secretion from airway epithelium leads to lung fibroblast proliferation and migration
Source: BMC Pulm Med. 2021 Nov 6;21:347. doi: 10.1186/s12890-021-01726-w (PMC8572483; doi:10.1186/s12890-021-01726-w)
Supplement: Supplementary file 4 — Additional file 4. Table S1: Characteristics of donors for primary fibroblasts isolation. [file 12890_2021_1726_MOESM4_ESM.docx]

| Supplementary table 1. Characteristics of donors for primary fibroblasts isolation | | | | | | |
| --- | --- | --- | --- | --- | --- | --- |
| Patients | Age, years | Sex | Smoking  status | FEV_1_ of %Pred | FEV_1_/FVC, % | Cancer types |
| 1# | 58 | F | No | 105.63 | 92.01 | Adenocarcinoma |
| 2# | 65 | M | No | 98.56 | 83.65 | Adenocarcinoma |
| 3# | 60 | M | No | 101.67 | 89.23 | Small-cell lung cancer |
| 4# | 63 | F | No | 97.22 | 86.36 | Adenocarcinoma |
| 5# | 67 | M | No | 95.25 | 79.28 | Adenocarcinoma |
| F, female; M, male; FEV1, forced expiratory volume in 1; FVC, forced vital capacity. | | | | | | |
